# Rapid evolution of post-zygotic reproductive isolation is widespread in Arctic plant lineages

**DOI:** 10.1093/aob/mcab128

**Published:** 2021-10-13

**Authors:** A Lovisa S Gustafsson, Galina Gussarova, Liv Borgen, Hajime Ikeda, Alexandre Antonelli, Lucas Marie-Orleach, Loren H Rieseberg, Christian Brochmann

**Affiliations:** 1 Natural History Museum, University of Oslo, Oslo, Norway; 2 Botany Department, Faculty of Biology and Soil Science, St Petersburg, Russia; 3 Tromsø University Museum, University of Tromsø, Tromsø, Norway; 4 Institute of Plant Science and Resources, Okayama University, Okayama, Japan; 5 Royal Botanic Gardens, Kew, Richmond, UK; 6 Gothenburg Global Biodiversity Centre, Department of Biological and Environmental Sciences, University of Gothenburg, Sweden; 7 Department of Plant Sciences, University of Oxford, Oxford, UK; 8 ECOBIO—Écosystèmes, Biodiversité, Évolution, Rennes, France; 9 Botany Department, University of British Columbia, Vancouver, Canada

**Keywords:** Arctic plants, hybrid sterility, incipient speciation, reproductive isolation

## Abstract

**Background and Aims:**

The Arctic tundra, with its extreme temperatures and short growing season, is evolutionarily young and harbours one of the most species-poor floras on Earth. Arctic species often show little phenotypic and genetic divergence across circumpolar ranges. However, strong intraspecific post-zygotic reproductive isolation (RI) in terms of hybrid sterility has frequently evolved within selfing Arctic species of the genus *Draba*. Here we assess whether incipient biological species are common in the Arctic flora.

**Methods:**

We conducted an extensive crossing experiment including six species representing four phylogenetically distant families collected across the circumpolar Arctic. We crossed conspecific parental populations representing different spatial scales, raised 740 F_1_ hybrids to maturity and measured fertility under laboratory conditions. We examined genetic divergence between populations for two of these species (*Cardamine bellidifolia* and *Ranunculus pygmaeus*).

**Key Results:**

In five of the six species, we find extensive reduction in pollen fertility and seed set in F_1_ hybrids; 219 (46 %) of the 477 F_1_ hybrids generated between parents separated by ≥427 km had <20 % pollen fertility. Isolation with migration (IM) and *BEAST analyses of sequences of eight nuclear genes in *C. bellidifolia* suggests that reproductively isolated populations of this species diverged during, or even after, the last glaciation. Likewise, Arctic populations of *R. pygmaeus* were genetically very similar despite exhibiting strongly reduced fertility in crosses, suggesting that RI evolved recently also in this species.

**Conclusion:**

We show that post-zygotic RI has developed multiple times within taxonomically recognized Arctic species belonging to several distantly related lineages, and that RI may have developed over just a few millennia. Rapid and widespread evolution of incipient biological species in the Arctic flora might be associated with frequent bottlenecks due to glacial cycles, and/or selfing mating systems, which are common in the harsh Arctic environment where pollinators are scarce.

## INTRODUCTION

The plants inhabiting the Arctic tundra must cope with extreme temperatures and variable and short growing seasons. Few species can tolerate such harsh conditions. Only about 1000 species regularly occur in the Arctic (Elven *et al.*, 2011), forming one of the most species-poor floras on Earth (Kier *et al.*, 2005). The Arctic tundra is young in evolutionary terms; it formed about 2–3 million years ago in response to climate cooling, mainly by immigration of plant lineages pre-adapted to cold climates (Murray, 1995; Brochmann *et al.*, 2013; Hoffmann *et al.*, 2017). Many of these species seem to have rapidly spread across the entire circumpolar region (Hultén, 1937; Brochmann *et al.*, 2013), and today the majority of Arctic species have vast, circumpolar ranges (Elven *et al.*, 2011). However, the current genetic structure in many circumpolar species appears to be mainly shaped by the last glacial cycle due to extensive regional extinction and recolonization, and the species often exhibit little phenotypic and genetic variation (Eidesen *et al.*, 2007; Skrede *et al.*, 2009; Ikeda *et al.*, 2017; unpubl. res.).

In its most classical definition, biological species are ‘groups of interbreeding natural populations that are reproductively isolated from other such groups’ (Mayr, 1942). Although the Arctic flora is poor in terms of taxonomically recognized species, there is some evidence that it may contain many incipient biological species, i.e. diverging populations showing at least some degree of reproductive isolation and thus representing early stages in the speciation process (see Coughlan and Matute, 2020, for a review). In three diploid Arctic species of the genus *Draba*, Grundt *et al.* (2006) found that partial to complete hybrid sterility has frequently evolved between conspecific populations, probably during the late Pleistocene. The results were based on intraspecific crosses at various spatial scales across the circumpolar Arctic and pollen fertility and seed set in first-generation hybrids. Only 8 % of the hybrids from crosses within and among geographic regions (Alaska, Greenland, Svalbard and Norway) were fully fertile. Hybrid sterility was positively correlated with genetic distance (*r* = 0.29–0.32), measured as Jaccard distance between amplified fragment length polymorphism (AFLP) genotypes (Grundt *et al.*, 2006). However, the lowest genetic distances for a population pair to reach 40 % hybrid sterility and 100 % hybrid sterility were 0.03 and 0.18, respectively. Intrinsic post-zygotic reproductive isolation (RI) was thus inferred to have evolved recently, but the genetic architecture underlying hybrid sterility was nevertheless complex and included Bateson–Dobzhansky–Muller (BDM) incompatibilities, single locus underdominance (i.e. a putative chromosomal translocation) and nuclear–cytoplasmic incompatibilities, inferred from genetic mapping and quantitative trait locus (QTL) analyses (Skrede *et al.*, 2008; Gustafsson *et al.*, 2014).

Hybrid sterility is probably mainly caused by the accumulation of BDM incompatibilities in geographically isolated populations (Dobzhansky, 1936; Muller, 1939, 1940), and it is often thought to develop very slowly (Hendry *et al.*, 2007). Levin (2012) surveyed dated plant phylogenies that include sister species for which data on the fertility of first-generation hybrids are available. He found that >4 million years of divergence is typically required for lineages to develop pronounced hybrid sterility. Similarly, Edmands (2002) reported that hybrid incompatibilities in diverging plant and animal lineages often begin to develop after hundreds of thousands of years, with partial compatibility persisting for >8 million years.

In a recent synopsis of intrinsic post-zygotic RI relative to genetic divergence in different taxonomic groups, Coughlan and Matute (2020) found large variation among species pairs in the level of divergence required to achieve complete RI. However, in most taxonomic groups, some species pairs achieved complete RI at low levels of divergence. In plants, there are some examples of potentially rapid evolution of hybrid sterility in addition to Arctic *Draba* (Grundt *et al.*, 2006). In the California endemic *Collinsia sparsiflora*, hybrid sterility between serpentine and non-serpentine ecotypes was found at small spatial scales and at low levels of genetic differentiation, suggesting recent divergence (Moyle *et al.*, 2012). Despite recent divergence, hybrid sterility has also been observed among three species in the *Mimulus tilingii* complex (Sandstedt *et al.*, 2021) and between *M. nasutus* and *M. guttatus* (Sweigart *et al.*, 2006); the latter two species were estimated to have diverged 200 000–500 000 years ago (Brandvain *et al.*, 2014). Strong reduction in hybrid fertility at low levels of genetic divergence has also been observed in orchids (Scopece *et al.*, 2008).

In their study of intrinsic post-zygotic RI in Arctic *Draba*, Grundt *et al.* (2006) only assessed hybrid sterility. Another potentially fast evolving mechanism conferring intrinsic post-zygotic RI in plants is hybrid seed failure (HSF) which results from developmental abnormality in the endosperm (e.g. Burkart-Waco *et al.*, 2015; Rebernig *et al.*, 2015; Baek *et al.*, 2016; Oneal *et al.*, 2016). After the onset of population divergence, HSF can develop rapidly as shown in *Mimulus* and *Solanum* (Lafon-Placette and Köhler 2016; Garner *et al.*, 2016; Roth *et al.*, 2018; Coughlan *et al.*, 2020). Crosses between the closely related *Arabidopsis lyrata* and *A. arenosa* mainly resulted in inviable seeds (Lafon-Placette *et al.*, 2017). In *Capsella*, different effective ploidy resulted in endosperm-based reproductive barriers (Lafon-Placette *et al.*, 2018). Rapid evolution of HSF has also been revealed among closely related species of *Ipomoea* (Ostevik *et al.*, 2020) and *Clarkia* (Briscoe Runquist *et al.*, 2014).

In this study, we address whether evolution of intraspecific post-zygotic RI is common in the Arctic flora, in spite of its recent origin and in spite of the low levels of phenotypic and genetic divergence observed within many species. It is possible that evolution of RI in the Arctic is accelerated by some particular characteristics, for example by frequent bottlenecks induced by the glacial cycles, and/or by selfing mating systems, which are common in the harsh Arctic environment where pollinators are scarce (Alsos *et al*., 2007, 2015; Brochmann *et al.*, 2013). We thus hypothesized that the Arctic flora may contain considerable cryptic diversity in terms of reproductively isolated populations that are included in the same taxonomic species based on morphology. To address this hypothesis, we collected living plants of 20 diploid species across the Arctic and cultivated them in a phytotron under simulated Arctic conditions. We successfully performed intraspecific crosses and measured F_1_ hybrid fertility for six of these species, representing four phylogenetically distant plant families, and we also investigated potential hybrid seed failure for two of them. We further selected two species to examine the relationship between RI and genetic divergence (*Cardamine bellidifolia* and *Ranunculus pygmaeus*).

## MATERIALS AND METHODS

### Study species

We collected 1711 living plants of 20 species representing 11 families across the circumpolar Arctic (Supplementary data Table S1). The species were selected according to four criteria. They should (1) be diploid, to avoid complexity associated with polyploidy; (2) reproduce sexually (i.e. no agamospermy reported); (3) be reported as common or widespread in the Panarctic Flora Checklist (Elven *et al.*, 2011), so that the species are easily located in the field; and (4) represent divergent phylogenetic lineages, to investigate whether the evolution of post-zygotic RI is common across diverse genera and families in the Arctic flora. The species were selected by consulting the Panarctic Flora Checklist (Elven *et al.*, 2011). Because Grundt *et al.* (2006) hypothesized that selfing may have accelerated the evolution of RI in Arctic *Draba*, we looked for suitable congeneric pairs of Arctic species reported with contrasting mating systems. We only found one such pair; the outcrossing *Silene acaulis* (L.) Jacq. and the predominantly selfing *S. uralensis* (Rupr.) Bocquet.

Cultivation in a phytotron and subsequent experiments were not successful for 14 species (the plants died or did not flower well in cultivation, or the crossing experiments failed; see Supplementary data Table S1), but we obtained data for the remaining six species: *Cardamine bellidifolia* L. and *Cochlearia groenlandica* L. (Brassicaceae); *Saxifraga hyperborea* R.Br. (Saxifragaceae); *Ranunculus pygmaeus* Wahlenb. (Ranunculaceae); and *Silene uralensis* and *Silene acaulis* (Caryophyllaceae).

These six species are all small, perennial herbs (*C. groenlandica* sometimes biennual). *Silene acaulis* is gynodioecious and predominantly outcrossing (Brochmann and Steen, 1999; Delph *et al.*, 1999), reported to be pollinated by bumble-bees in Canada (Colla and Dumesh, 2010) but likely to be pollinated by Diptera in Svalbard, where bumble-bees are absent (Brochmann and Steen, 1999). We found no specific information on potential pollinators of the five other species (most probably Diptera), which all have been reported to be predominantly selfing (Tikhmenev, 1984; Molau, 1993; Brochmann and Steen, 1999; Brochmann and Håpnes, 2001; Elven, 2005). In terms of morphology, all six species are typical short-distance seed dispersers, either via rapid opening of the fruit (*C. bellidifolia*) or via wind-induced movement of dry infructescences. Long-distance dispersal is nevertheless likely to occur with wind across snow and ice and with sea currents, as suggested for many Arctic plant species (Abbott and Brochmann, 2003; Brochmann *et al.*, 2003; Alsos *et al*., 2007, 2015). Detailed information of these six species is provided in the Supplementary data Appendix S1, and data reported from here on are only referring to these six species.

### Field sampling and cultivation

We collected plant material from three regions: Alaska/Yukon; the North Atlantic and high-Arctic archipelago of Svalbard; and mainland Norway, and from four sub-regions within the Alaska/Yukon region (Supplementary data Table S2). Pairwise geographic distances between collection sites from different regions were 3445–4038 km (Alaska/Yukon–Svalbard), 5258–5877 km (Alaska/Yukon–mainland Norway) and 1860 km (Svalbard–mainland Norway); and 427–896 km between sites from different sub-regions in Alaska/Yukon (Supplementary data Table S3). We defined one population as plants occurring within an area of 100 m × 100 m. Individual plants were collected at least 10 m apart, except for a few populations, which were restricted to small areas (i.e. *C. bellidifolia*: populations LG09-S-32, LG09-A-104, LG09-N-130; *C. groenlandica*: population LG09-S-24, LG09-A-48; *S. hyperborea*: population LG09-S-23, LG09-A-103; and *R. pygmaeus*: population LG09-A-47, LG09-A-102). From most populations, we collected ten living plants, one plant as a voucher, and leaves from five plants dried in silica gel. The roots were cleaned for soil and whole plants were wrapped in moist paper and put in plastic bags for shipping to Norway. The plants were replanted and cultivated for two flowering seasons per year in a phytotron without any pollinating insects at the Department of Biosciences, University of Oslo, Norway (cultivation conditions as specified in Brochmann *et al.*, 1992).

### Flow cytometric verification of homogeneity in ploidy level

To ensure ploidy homogeneity among populations, we determined relative fluorescence intensities of intact nuclei isolated from silica-dried leaf tissue using 4′,6-diamino-2-phenylindole (DAPI) flow cytometry (Dolezel *et al.*, 2007). In total, 182 plants were analysed (for details, see Supplementary data Table S4). We selected a two-step simplified procedure using Otto buffers, and fresh material of *Solanum pseudocapsicum* (2C = 2.59 pg; Temsch *et al.*, 2010) served as a primary internal reference standard (with genome size close to, but not overlapping with, that of most analysed species). *Glycine max* ‘Polanka’ (2C = 2.50 pg; Doležel *et al.*, 1994) and *Bellis perennis* (2C = 3.38 pg; Schönswetter *et al.*, 2007) served as additional internal standards for *R. pygmaeus* and *S. acaulis*, respectively. We recalculated the recorded fluorescence values for the latter two species with *Solanum* as a standard. Ten per cent of the samples were analysed twice to assess the between-run fluorescence stability.

### Crossing experiments

To evaluate the relationship between geographic distance and accumulation of intrinsic post-zygotic RI, we designed a crossing programme covering three scales: within-population crosses (WPCs; functioning as positive controls), within-region crosses (WRCs) and across-region crosses (ARCs). Except for *C. groenlandica*, WRCs were performed among the four sub-regions in the Alaska/Yukon region: Seward Peninsula; Brooks Range; Central Alaska along Denali Highway; and Yukon Territory. *Cochlearia groenlandica*, which is primarily a coastal species, was only collected in one of these sub-regions (Seward Peninsula), and we used different populations from this sub-region for WRCs (populations separated by 46–98 km). Therefore, the geographic distance between these WRC populations was shorter than for the other species (see Supplementary data Table S3). ARCs were performed among all three regions (Alaska/Yukon, Svalbard and mainland Norway). Large parts of the Svalbard archipelago are difficult to access, and we were only able to collect populations separated by 4–10 km in this region. Crosses performed among these closely situated populations are referred to separately as Svalbard population crosses (SPCs).

A total of 199 parental plants from 36 populations were successfully used in the crossing experiments, including ten populations (64 plants) of *C. bellidifolia*, six populations (43 plants) of *C. groenlandica*, five populations (41 plants) of *S. hyperborea*, six populations (27 plants) of *R. pygmaeus*, two populations (two plants) of *S. uralensis* and seven populations (22 plants) of *S. acaulis* (Supplementary data Tables S2 and S3). In each crossing experiment, we emasculated (i.e. removed all stamens from) 1–8 flower buds on the maternal plant 1–4 days before anthesis to avoid self-fertilization, except for *S. acaulis*, which is gynodioecious, for which we selected female plants. We transferred pollen 2–9 d later from a selected paternal plant to the emasculated flowers, depending on timing of stigma receptivity. Whenever possible, we performed reciprocal crosses. Seeds were harvested at maturity and stored for vernalization for 3–5 months at 4 °C before sowing.

### Hybrid seed failure analysis

We selected *C. bellidifolia* and *C. groenlandica* for investigation of potential hybrid seed failure, using the potential maximum seed set in cultivated parental plants as a reference. To estimate the potential maximum seed set, we counted the total number of ovules in 292 fruits from 65 individuals of *C. bellidifolia* and in 148 fruits from 40 individuals of *C. groenlandica*, calculated the average per fruit per individual and then averaged over individuals. We found the average number of ovules per fruit, i.e. the potential maximum seed set, to be 11 in *C. bellidifolia* and 12 in *C. groenlandica*. We calculated the percentage seed set after a cross as the number of fully developed, well-formed seeds relative to potential maximum seed set (Supplementary data Table S3). We also recorded germination success for *C. bellidifolia* and *C. groenlandica*, but the scope of this study did not allow for examination of endosperm development.

To test the effects of cross type (i.e. WPC, SPC, WRC and ARC) on seed set and germination success, we ran generalized linear mixed models (GLMMs) for each species separately using the lme4 R package (Bates *et al.*, 2015). We first fitted GLMMs by maximum likelihood using Laplace approximation, with a binomial error distribution, including cross type as a fixed effect, and maternal population and paternal population as random effects. We estimated overdispersion with the blmeco R package (Korner-Nievergelt *et al.*, 2015), and consequently accounted for overdispersion in all GLMMs by including an observation-level random effect to provide more accurate parameter estimates (see Harrison, 2015). We then estimated the cross type effects by performing likelihood ratio tests (LRTs; which is an often used statistical procedure to test the significance of either fixed or random effects in GLMMs, e.g. Arbuthnott *et al.*, 2014; Wright *et al.*, 2020). These LRTs test the significance of a focal variable (here, cross type) by determining whether the goodness of fit of the model decreases after excluding the focal variable from the explanatory variables of the model. If removing cross type from the model induces a significant decrease of the goodness of fit of models predicting hybrid fitness, we can conclude that cross type is a significant predictor of hybrid fitness. For practical reasons, we were unable to assess seed production and germination success for the other species (*S. hyperborea*, *R. pygmaeus*, *S. uralensis* and *S. acaulis*).

### Fertility of intraspecific F_1_ hybrids and parental populations

F_1_ seeds from the experimental crosses in *C. groenlandica* and *S. acaulis* were scarified with fine sandpaper prior to sowing in order to break their rigid seed coat; seeds of the other species were sown directly. Depending on species, seed availability and germination success, 1–14 (average four) F_1_ seedlings from each cross were raised to maturity (see Supplementary data Table S3 for details). We estimated experimental F_1_ hybrid fertility as the percentage of stainable pollen and as the percentage of seed set. Pollen stainability was estimated by counting the proportion of fully stained pollen grains after adding lactophenol in cotton blue to pollen transferred to a microscope slide; on average, 223 pollen grains were counted for each plant (cf. Brochmann *et al.*, 1993).

To obtain seed set in the F_1_ hybrids of *S. acaulis*, we transferred pollen from hermaphroditic to female plants among the F_1_ hybrids obtained from the same cross. The other five species spontaneously self-pollinated in the phytotron, and for these species we let the F_1_ hybrid spontaneously self and calculated seed set by opening the fruit, counting the total number of ovules (i.e. the sum of apparently undeveloped ovules, poorly developed seeds and fully developed seeds) and calculating the percentage of fully developed seeds relative to the total number of ovules. Fruits were sampled randomly and 1–12 (average four) fruits were examined per F_1_ hybrid. We selected this procedure for estimating seed set in F_1_ hybrids for practical reasons, and realize that in many cases, the poor pollen fertility of many hybrids would necessarily lead to poor seed set after selfing and thus not accurately reflect the female fertility of the hybrid.

The fertility of a total of 177 plants from the parental populations was also estimated as described above (Supplementary data Table S5) and included as positive controls. Most of the parental plants used in the crossing experiments were estimated for fertility, but some had died at the time of phenotyping.

We hereafter refer to pollen stainability as pollen fertility. It follows from the above description that pollen fertility and seed set are not independent measurements of RI.

### Statistical analysis on experimental F_1_ hybrids

We analysed the F_1_ pollen fertility and seed set data for each species separately. First, we tested for correlations between F_1_ pollen fertility and seed set by performing a Spearman correlation. Second, we tested for an effect of cross type [i.e. WPC, SPC, WRC and ARC, as well as parental population (PP, as positive control) pollen fertility and seed set data] by using GLMMs (fitted by maximum likelihood using Laplace approximation), with binomial error distribution, including cross type as fixed effect, and maternal population, paternal population and F_1_ family as random effects. We accounted for overdispersion by adding an observation-level random effect (Harrison 2015), and then performed LRTs to assess the cross-type effect. When the effect of cross type was significant, we performed post-hoc Tukey pairwise comparisons between cross types by using the emmeans R package (Lenth, 2020). Note that the model failed to converge at times (probably due to overfitting issues). When this occurred, we simplified our model by removing random effects that explained little variance (see Supplementary data Table S6 for final models used). This procedure, however, did not succeed for seed set in *S. hyperborea*, for which we therefore report outcomes from the model not accounting for overdispersion (dispersion factor: 2.33). Third, we tested for the effects of geographic distance between parental populations on F_1_ pollen fertility and seed set by using a similar statistical procedure as for cross type, but using distance as a fixed effect instead of cross type, and excluding PP pollen fertility and seed set data. To limit scale difference between variables, distance was square root transformed.

In addition, we analysed F_1_ pollen fertility and seed set data from the reciprocal crosses to infer the mode of F_1_ incompatibility (i.e. if hybrid incompatibility is due to nuclear–nuclear incompatibilities and/or cytonuclear incompatibilities). Reciprocal crosses yielded two levels of relatedness: parafamily (i.e. individuals sharing the same parents, regardless of the direction of the cross) and family (i.e. individuals sharing the same mother and the same father). We tested if family provided additional information relative to parafamily by using LRTs comparing the goodness of fit of models with and without the family explanatory variable. We first built GLMMs (fitted by maximum likelihood with Laplace approximation) with binomial error distribution, including cross type as fixed effect, and maternal population, paternal population, parafamily and the observation level as random effects. We then built the same model, including family as an additional random effect, and compared the goodness of fit of the two models. If the model including family provides a better fit, then F_1_ incompatibility depends on the direction of the cross – suggesting that the incompatibility is partially caused by cytonuclear incompatibilities and/or genomic imprinting. When the model failed to converge, we removed the maternal population and/or the paternal population random effects in both models (i.e. the model with and without the family term).

### Molecular analysis of Cardamine bellidifolia

We selected this species for estimation of divergence time between populations because outgroup data and primers for sequencing nuclear genes were available from our previous study (Ikeda *et al.*, 2012). In the present study, we inferred its phylogenetic and demographic history based on sequencing of eight nuclear genes (*CHS*, *CO*, *COP1*, *DET1*, *DFR*, *F3H*, *FRI* and *GA1*). DNA was extracted from fresh leaves of cultivated plants that were raised from seeds harvested from spontaneously selfed parental plants from eight sites (Supplementary data Table S2) using the DNeasy Plant Mini Kit (Qiagen) according to the manufacturer’s instructions. We sequenced two individuals from each site (except only one from Nome) using the same primers as Ikeda *et al.* (2012), as well as newly designed primers for this study (FRI_F: GCAGTGGAAACATTCAAACGCCA and FRI_R: CAGGCATTAGAAGAAAAGACTCCAG). PCR was performed following Ikeda *et al.* (2012), and sequences of each locus were directly determined using an ABI 3130-avant Genetic Analyzer (POP-7 polymer and 36 cm capillary; Applied Biosystems). Haplotype sequences of each locus were inferred by Bayesian statistical methods using PHASE (Stephens *et al.*, 2001; Stephens and Donnelly, 2003). The number of segregating sites (*S*) and pairwise differences (*π*; Tajima, 1983) within *C. bellidifolia* were calculated for each locus using POPGENOME (Pfeifer *et al.*, 2014). For estimating genetic distance between individuals within populations and between populations, we calculated the average number of nucleotide differences (Wakeley, 1994) using POPGENOME (Pfeifer *et al.*, 2014).

Phylogenetic relationships among samples of *C*. *bellidifolia* originating from different regions (mainland Norway, Svalbard, Alaska and Yukon) were inferred using *BEAST (Heled and Drummond, 2010), including its sister species, the Japanese alpine endemic *C. nipponica* (Ikeda *et al.*, 2012), as well as *C. alpina* (two specimens), *C. resedifolia* (two specimens) and *C. glauca* (one specimen) as outgroups. For the outgroup plants, we used published sequences from Ikeda *et al.* (2012) except that *FRI* was sequenced for this study. The best fitting substitution model for each of the eight nuclear loci was selected using jModeltest2 under the Bayesian information criterion (Darriba *et al.*, 2012). In preliminary analyses, all combinations of the prior speciation process (the Birth–Death or the Yule speciation model), population size (constant, linear or constant and linear model) and two clock models (strict clock or lognormal relaxed clock) were assumed, for which Markov chain Monte Carlo (MCMC) searches were run for 3.0 × 10^8^ iterations, sampling every 10 000th iteration. After discarding 3.0 × 10^7^ iterations as the burn-in and checking the convergence of analyses by calculating effective sample sizes (ESS >200 for important parameters such as likelihood), each model was assessed against the others using the approximate Bayes Factor (BF) comparison (Kass and Raftery, 1995) implemented in Tracer ver. 1.5 (Rambaut and Drummond, 2009). Since no model was better than the simplest one assuming a Yule speciation prior, constant population sizes and a strict clock (2lnBF <10), the final tree was estimated by assuming these settings with four independent MCMC searches run under the same conditions as the preliminary analysis. After discarding 5.0 × 10^7^ iterations as the burn-in and checking the convergence for each MCMC search, all runs were combined into a single file using LogCombiner ver. 1.7 (Drummond and Rambaut, 2007). A maximum clade credibility tree was obtained in TreeAnnotator ver. 1.4 (Rambaut and Drummond, 2008). Individual gene trees were visualized using DensiTree (Kass and Raftery, 1995) and summarized as a maximum clade credibility tree with a posterior probability limit of 0.95. In order to assess the informativeness of the data and influence of prior parameters on posterior values, the analysis was also run with an empty alignment, i.e. sampling from priors only.

We assessed the timing of the divergence between plants from each pair of geographical regions (Alaska/Yukon, Svalbard and mainland Norway) for *C. bellidifolia*. Four individuals from Brooks Range were used as representatives of Alaska/Yukon. First, we estimated demographic history under the isolation with migration (IM) model using the program IMa2 (Hey, 2010). Probability density functions for demographic parameters (population size: *θ*_0_, *θ*_1_, ancestral population size: *θ*_A_, migration rates between pairs of populations: *m*_0_ >1, *m*_1_ >0, and divergence time *t*) were estimated by 1.0 × 10^7^ MCMC steps following 1.0 × 10^5^ burn-in periods with ten chains under Metropolis coupling. After obtaining probability density functions from three replicate runs, the maximum likelihood estimates (MLEs) and highest posterior densities (HPDs) were estimated based on these functions. Divergence times were scaled by the mutation rate used in previous studies [8.67 × 10^−9^ substitutions per site year^–1^ (ssy^–1^); Ikeda *et al*., 2009, 2012], which was estimated based on synonymous mutation rates of the *CHS* gene in Brassicaceae (1.5 × 10^−8^ substitutions per site per year; Koch *et al.*, 2001). In addition, the divergence time of plants from the same pairs of geographic regions was estimated based on the time of their most recent common ancestor (TMRCA) in the *BEAST analysis and summarized using Tracer ver. 1.5 (Rambaut and Drummond, 2009). The time was scaled by assuming mutation rates for each gene as normal distributions (mean 7.1 × 10^–9^ ssy^–1^ with s.d. 7.0 × 10^–10^ ssy^–1^) based on the spontaneous mutation rate calculated in an experimental study of *Arabidopsis thaliana* (Ossowski *et al.*, 2010).

### Molecular analysis of Ranunculus pygmaeus

We selected *R. pygmaeus* as a second species for molecular analysis, because we had additional material available and previous experience with AFLP analysis of this species (Schönswetter *et al.*, 2006). Here, we added 34 new samples to the samples from Schönswetter *et al.* (2006) to assess relationships among our populations in the context of the broader history of the species. A total of 97 individuals and seven replicates were successfully analysed for AFLPs (for details, see Supplementary data Table S2). Total genomic DNA was extracted from silica gel-dried leaf material using the DNeasy Plant Mini Kit (Qiagen) according to the manufacturer’s instructions. Because we observed very limited AFLP variation, we tested a total of 41 primer combinations. To increase the number of bands, we also tested primers with only two selective nucleotides. The three most informative primer combinations were chosen for further analysis (fluorescent dye in parentheses): EcoRI AGT (6-FAM)–MseI CGC; EcoRI ATG (VIC)–MseI CGA; and EcoRI ACC (NED)–MseI CGT. The AFLP procedure followed Gaudeul *et al.* (2000) with some modifications (Schönswetter *et al.*, 2006). For each individual, 2 µL of 6-FAM-, 2 µL of VIC- and 3 µL of NED-labelled selective PCR products were diluted in 14 µL of H_2_O of which 3.5 µL were mixed with 0.3 µL of GeneScan ROX 500 (Applied Biosystems) and 11.7 µL of formamide, and run on a capillary sequencer (ABI 3100, Applied Biosystems). Reproducibility was estimated as the average proportion of correctly replicated bands (Bonin *et al.*, 2004). Raw data were imported and scored using GeneMapper ver. 3.7 (Applied Biosystems) and exported as a presence/absence matrix.

To visualize the main structure in the data, we performed a principal co-ordinate analysis (PCoA) in NTSYS-PC ver. 2.02h (Rohlf, 1990) using Dice’s coefficient of similarity. We estimated genetic diversity using the R-script AFLPdat (Ehrich, 2006) according to Nei’s formula for gene diversity (Nei, 1978), i.e. as the average proportion of differences between pairs of individuals. We performed a Bayesian analysis of the genetic data using MrBayes ver. 3.2.2 (Ronquist and Huelsenbeck, 2003). We ran two independent analyses with 20 million generations each, sampling every 1000th generation, employing three heated chains and one cold chain, and coding the AFLP data as ‘lset coding=variable’ following the user’s manual. We ensured that the analyses reached convergence (the average standard deviations of split frequencies were far below 0.01) and excluded 5000 trees from each run as an initial burn-in phase. We then computed a 50 % majority-rule consensus phylogram with branch lengths proportional to mean lengths out of the entire post-burn-in sample, based on a total of 30 002 trees.

## RESULTS

### Flow cytometric verification of homogeneity in ploidy level

Histograms with distinct peaks attributable to the internal standard and target species were detected in all 182 samples analysed by flow cytometry (Supplementary data Fig. S1; Table S4). Coefficients of variation of reference standard peaks did not exceed 3 %; coefficients of variation of sample peaks varied from 1.82 to 8.75 % (mean 5.32 %), depending on the species. Except for one individual of *S. hyperborea* and one of *S. uralensis* that we found to be polyploid and therefore excluded from the crossing experiments, fluorescence intensities of all parental plants were uniform both within and among geographic regions (Supplementary data Table S4). Considering that only diploid chromosome counts have been reported for the analysed species (see Supplementary data Appendix S1), we conclude that our flow cytometric analyses confirmed diploidy in all but two samples.

### Hybrid seed failure

We observed considerable variation among developing seeds, ranging from apparently undeveloped ovules to various sizes and degrees of misformed seeds, realizing that some of the apparently undeveloped ovules might have been unfertilized ovules (and thus represent a post-mating, pre-zygotic barrier) rather than hybrid seed failure resulting from maladaptive interactions between the zygote and the endosperm. Since we were not able to distinguish between these alternatives, we might have overestimated hybrid seed failure. However, we found no effects of cross type on seed set and germination success for the two species investigated (*C. bellidifolia* and *C. groenlandica*; LRTs *P-*values >0.05; Supplementary data Table S7).

### Fertility of intraspecific F_1_ hybrids and parental populations

**Fig. 1.**
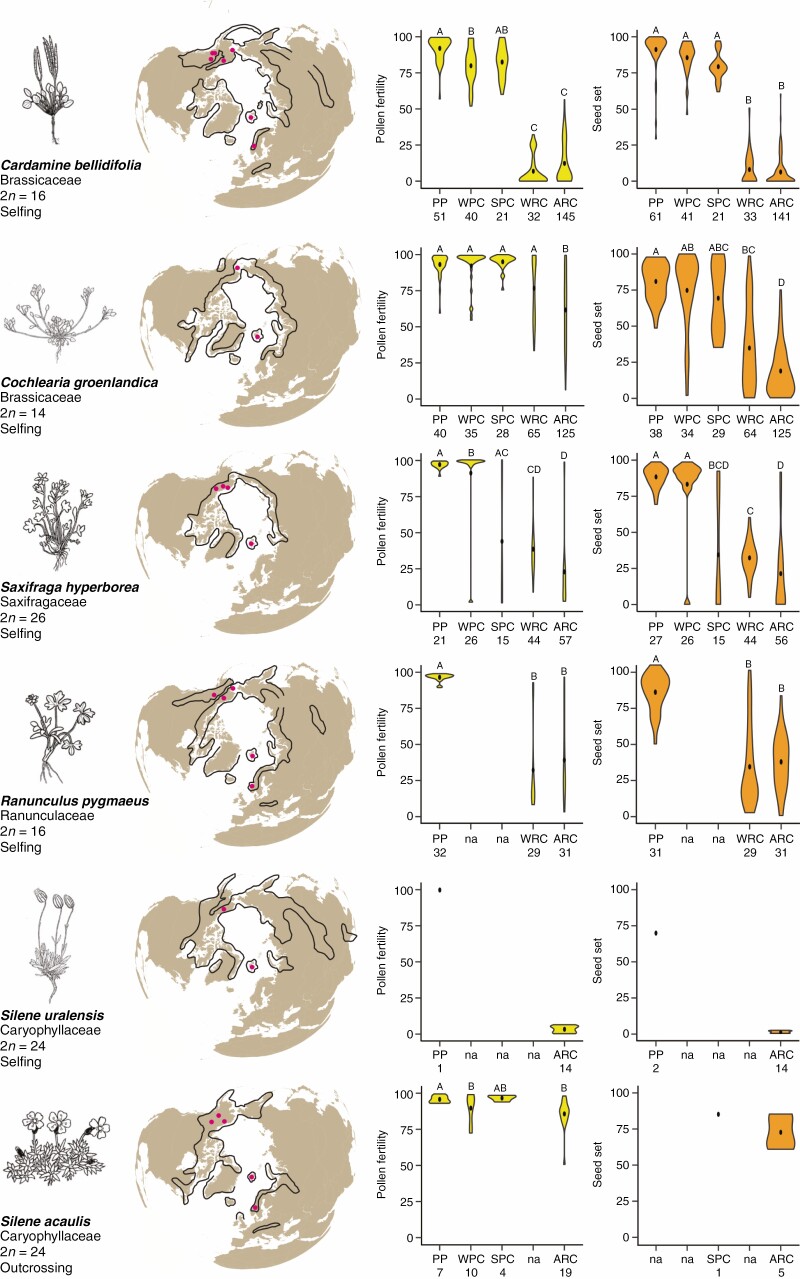
Pollen fertility (yellow) and seed set (orange) in parental plants and experimental intraspecific F_1_ hybrids in six Arctic diploid species. Maps show total geographic range (lines) and sampling locations (red dots, see Supplementary data Table S2). Pollen fertility was estimated by counting the proportion of fully stained pollen grains, and seed set as the proportion of fully developed seeds relative to the total number of ovules (note that seed set in the five selfing species was assessed after spontaneous selfing; see the Materials and Methods for details). In the violin plots, the median is shown as a black dot, and letters above indicate significance of differences among cross types (*P-*value <0.05) determined by post-hoc Tukey pairwise comparisons. PP, parental populations; cross types are WPCs, within-population crosses; SPCs, Svalbard-population crosses; WRCs, within-region crosses; ARCs, across-region crosses. SPC is shown separately because these populations were collected from closely adjacent sites. WRCs were performed among four sub-regions in the Alaska/Yukon region (Seward Peninsula, Brooks Range, Central Alaska along Denali Highway, and Yukon Territory), except for *Cochlearia groenlandica*, which is a coastal species only collected in Seward Peninsula. ARCs were performed between the three main geographic regions (Alaska/Yukon, Svalbard and mainland Norway). Sample sizes (number of plants) are provided for each cross type.

The 177 plants from the field-collected parental populations showed, with a few exceptions, high pollen fertility and seed set (Fig. 1; Supplementary data Table S5). In total, 201 crosses produced F_1_ hybrids that appeared to be fully viable, and 740 F_1_ hybrids were analysed for pollen fertility and 709 for seed set (Supplementary data Table S3). Pollen fertility and seed set were positively correlated in *C. bellidifolia* (*r*_S_ = 0.890), *C. groenlandica* (*r*_S_ = 0.749), *S. hyperborea* (*r*_S_ = 0.887) and *R. pygmaeus* (*r*_S_ = 0.861; Spearman’s rank correlation coefficient; *P* < 0.0001 for all species); we did not obtain sufficient data for *S. uralensis* and *S. acaulis* to test for this correlation.

### Effects of cross type on hybrid incompatibilities

We found strong evidence for widespread hybrid incompatibilities (Fig. 1). The effect of cross type (i.e. WPC, SPC, WRC and ARC, as well as PP pollen fertility and seed set) was significant for pollen fertility for all species, and for all species but *S. acaulis* for seed set (we obtained too few crosses in *S. uralensis* to test for effects of cross type; LRTs *P*-values <0.05; Supplementary data Table S6). We did not succeed in performing WPCs and SPCs for *R. pygmaeus* and WRCs for *S. acaulis*. Overall, in the following post-hoc pairwise comparisons to test which cross-type pairs were different from each other, crosses both within (WRCs) and among geographic regions (ARCs) resulted in hybrids with significantly reduced fertility relative to PP, SPCs and WPCs (see Fig. 1 and Supplementary data Table S8 for details). We saw two notable deviations from this pattern: in *C. groenlandica*, WRC did not differ from PP and WPC for pollen fertility and from WPC for seed set; however, for this species, the populations used for WRC were geographically closer to each other than for the other species; and, in *S. acaulis*, the only significant cross-type pair comparison was between PP and ARC and between PP and WPC (Fig. 1; Supplementary data Table S8).

### Hybrid incompatibilities and geographic distance

The effect of geographic distance between the parental populations on F_1_ pollen fertility and seed set provided generally consistent outcomes, i.e. overall, F_1_ hybrid fitness decreased with increasing distance. Distance had significant effects on pollen fertility and seed set in *C. bellidifolia*, *C. groenlandica* and *S. hyperborea* (LRTs *P*-values <0.005), but not for *R. pygmaeus* (LRTs *P*-values >0.05; however, we did not have WPC and SPC values for this species) and *S. acaulis* (LRTs *P*-values >0.05; too few crosses were obtained in *S. uralensis* to test for distance effects; Supplementary data Table S9).

### Reciprocal crosses

The analyses of the reciprocal crosses, which were performed for three of the species, suggested that the direction of the cross significantly affected F_1_ pollen fertility and seed set for *C. groenlandica* and *S. hyperborea*, but not for *C. bellidifolia* (Supplementary data Table S10; Fig. S2). We were unable to fit the statistical model for seed set in *C. bellidifolia*, and we therefore cannot show statistical outcomes for seed set of this species, but visual inspection suggests no effect of the direction of the cross (Supplementary data Fig. S2).

### Molecular analysis of Cardamine bellidifolia

**Fig. 2.**
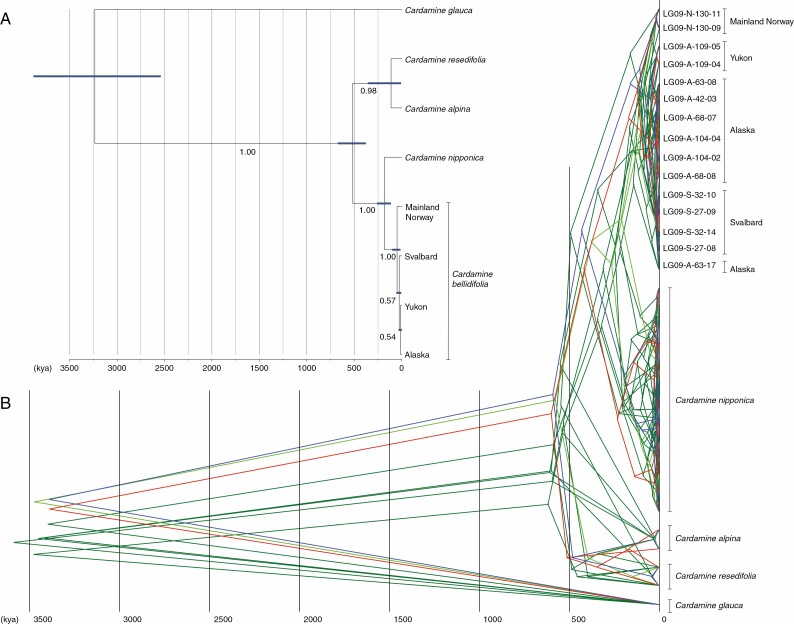
(A) Species tree of *Cardamine bellidifolia* and its relatives estimated using *BEAST. Bars on the internal nodes represent the 95 % HPD of the node ages, and the numbers along branches are Bayesian posterior probabilities. (B) Individual gene trees of *C. bellidifolia* and its relatives summarized as a maximum clade credibility tree with a posterior probability limit of 0.95. Different colours represent individual gene trees. Scales are in thousands of years (kya).

We found virtually no phylogenetic resolution among reproductively isolated populations in *C. bellidifolia* based on sequencing of eight nuclear genes (Fig. 2; see Supplementary data Table S11 for statistics of analysed loci). The lowest genetic [average pairwise nucleotide diversity per site (Pi)] distance for a population pair to reach 100 % reproductive isolation (hybrid sterility) was only 0.0005 (Supplementary data Fig. S3). In the IM analysis, the estimated demographic parameters for each pair of geographic regions were consistent among three independent replicates, and an unambiguous peak of posterior probability was obtained for each parameter (Supplementary data Fig. S4; Table S12). We therefore considered the results to be robust. Divergence between Alaska and Svalbard was estimated to be the most recent (2600 years before present, BP; 95 % HPD 0–35 600 years BP), and divergence between Alaska and mainland Norway was estimated to be the oldest (22 000 years BP, 95 % HPD 3000–186 600 years BP; Table 1; Fig. 2; these results may, however, be somewhat biased because of shallow divergence, cf. Cruickshank and Hahn 2014).

**Table 1.** Estimates of divergence time (years) between populations of *Cardamine bellidifolia* separated by post-zygotic isolation barriers

Pairs of geographic regions	IMa		*BEAST		
	MLE	95 % HPD	Mean	Median	95 % HPD
Alaska vs. Svalbard	2573	(0–35 563)	30 693	28 330	(4366–61 667)
(pop0 = LG09-A-63 + LG09-A-68, pop1 = LG09-S-27 + LG09-S-32)					
Mainland Norway vs. Alaska	21 951	(3046–186 638)	50 210	46 465	(8395–99 459)
(pop0 = LG09-N-130, pop1 = LG09-A-63 + LG09-A-68)					
Mainland Norway vs. Svalbard	8115	(866–150 067)	49 553	45 670	(7414–98 333)
(pop0 = LG09-N-130, pop1 = LG09-S-27 + LG09-S-32)					

Estimates are based on the isolation with migration model (IMa) and on the time since the most recent common ancestor in Bayesian analysis of the species tree (*BEAST) using sequences of eight nuclear genes (see the Materials and Methods and Supplementary data Table S1)

Also in the *BEAST analysis, the most recent population split was estimated between Alaska and Svalbard (TMRCA: mean 30 700 years BP, 95 % HPD 4400–61 700 years BP), and the oldest between mainland Norway and the other areas (mean 50 200 vears BP, 95 % HPD 8400–99 500 years BP; Table 1). The differences between these analyses may partly have been caused by the variation in posterior estimates of the mutation rate from a prior setting in *BEAST, whereas a fixed value was provided in the IM analysis based on synonymous mutation rates in Brassicaceae (Koch *et al.*, 2001).

### Molecular analysis of Ranunculus pygmaeus

**Fig. 3.**
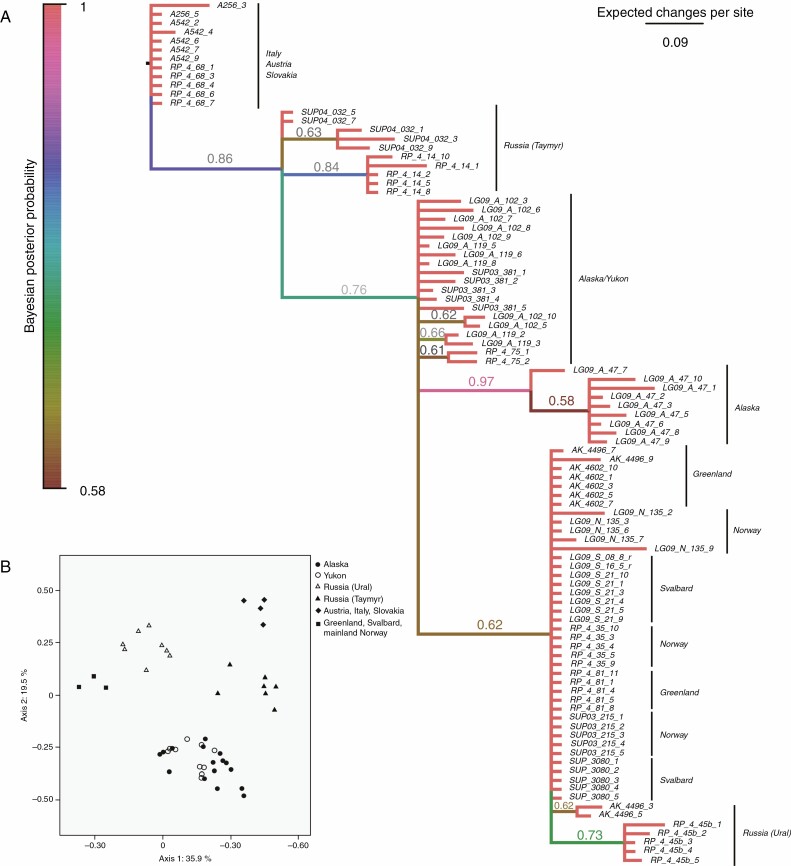
(A) Bayesian phylogram of AFLP phenotypes of *Ranunculus pygmaeus*, inferred with MrBayes. Branch lengths are mean lengths estimated from 30 002 trees after an initial burn-in phase of 10 000 trees. Numbers above branches are Bayesian posterior probabilities (only those >0.5 are shown). Samples beginning with ‘LG09’ indicates those samples used in our crossing experiments, whereas the remaining samples were re-analysed from Schönswetter *et al.* (2006). (B) Principal co-ordinate analysis of AFLP phenotypes observed in *R. pygmaeus* based on Dice’s coefficient of similarity. The percentage of variation explained by each axis is indicated. Symbols identify the main geographic regions.

Among the reproductively isolated populations of *R. pygmaeus*, we also observed little and poorly structured genetic variation, with a final AFLP dataset including only 34 polymorphic markers (reproducibility 99.16 %). Virtually no variation was observed in the northern North Atlantic area. The MrBayes tree (Fig. 3) revealed limited phylogeographic structure, with low support values except for a clade unifying some of the Alaskan samples (Bayesian posterior probability = 0.97). The majority of the samples from Alaska and Canada (Yukon) did not attain any supported groupings. The populations from the Alps and the Tatra Mountains formed a clade, followed by Russian (Taymyr) samples. The Greenland, Norway and Svalbard populations formed a poorly supported clade, comprising a separate clade of samples from the Ural Mountains in Russia. The PCoA plot (Fig. 3) revealed some geographic structuring that was largely consistent with the MrBayes tree.

## DISCUSSION

### Rapid evolution of post-zygotic reproductive isolation is widespread in Arctic plants

We have demonstrated that evolution of intraspecific intrinsic post-zygotic RI conferred by hybrid sterility is not limited to the three Arctic species of *Draba* investigated in previous studies in our laboratory (Grundt *et al.*, 2006; Skrede *et al*., 2008, 2009; Gustafsson *et al.*, 2014). We found that strong hybrid incompatibilities have also developed within five (of the six examined) Arctic species belonging to other genera of the Brassicaceae (*Cardamine bellidifolia* and *Cochlearia groenlandica*) as well as to other, distantly related plant families: Saxifragaceae (*Saxifraga hyperborea*), Ranunculaceae (*Ranunculus pygmaeus*) and Caryophyllaceae (*Silene uralensis*; Fig. 1). Remarkably, most populations examined of each of these five taxonomic species as well as of the three examined by Grundt *et al.* (2006) have accumulated hybrid incompatibilities and can therefore be considered to represent incipient species. Future studies are needed to address potential morphological differentiation among these populations, but it is notable that no or only little intraspecific morphological variation has been reported within these species in the taxonomic literature (summarized in Supplementary data Appendix S1; cf also Elven *et al.*, 2011).

While the parental plants as well as the F_1_ hybrids from intrapopulation crosses were highly fertile, we found that the F_1_ hybrids from within- and across-region crosses in these species showed significant reductions in pollen fertility and seed set (except for within-region crosses in *C. groenlandica*, for which the parental populations were closely situated; too limited data were available for *S. uralensis*; Fig. 1; Supplementary data Tables S6 and S8). For two species (*C. groenlandica* and *S. hyperborea*), we also found evidence for cytonuclear incompatibilities based on reciprocal crosses (Supplementary data Table S7; Fig. S2). It is therefore likely that the reduction in fertility is caused by incompatibilities both among nuclear genes and between nuclear and cytoplasmic genes, and possibly by chromosomal rearrangements. In agreement with the results for Arctic *Draba* (Grundt *et al.*, 2006), the reduction in hybrid fertility was significantly correlated with geographic distance in *C. bellidifolia*, *C. groenlandica* and *S. hyperborea* (but not in *R. pygmaeus*; however, the data for this species may have been too limited for detection of a geographical signal). Notably, we did not find evidence for hybrid seed failure, an intrinsic post-zygotic barrier reported to develop quickly in some plant lineages (e.g. Lafon-Placette and Köhler, 2016; Garner *et al.*, 2016; Roth *et al.*, 2018; Coughlan *et al.*, 2020), based on seed set and germination success in the two species examined (*C. bellidifolia* and *C. groenlandica*).

Our results thus suggest that incipient speciation is widespread in the Arctic flora. This is of particular interest because the Arctic flora is poor in terms of taxonomically recognized species (Kier *et al.*, 2005; Elven *et al.*, 2011), as expected given its position at the extreme end of the latitudinal diversity gradient. The results of this study stand in marked contrast to the findings of Levin (2012), who showed that pronounced hybrid sterility in plants typically needs >4 million years of divergence to develop between sister lineages. Although analytic and calibration uncertainties may to some degree have affected the estimation of divergence time in our multilocus sequence analyses of the reproductively isolated populations of *C. bellidifolia*, RI has obviously evolved very recently in this species, possibly during the last glacial period when it seems to have expanded across the entire circum-Arctic region (Fig. 2; Table 1). Moreover, populations of *R. pygmaeus* originating from different areas in Alaska/Yukon were genetically very similar despite exhibiting significantly reduced fertility in interpopulation crosses, suggesting that RI evolved recently in this species as well (Fig. 3). While reduced fertility in intraspecific crosses has been reported in taxa from other floras (reviewed in Rieseberg *et al.*, 2006), it appears to be infrequent and contrasts sharply with what we have reported here for Arctic plants. Overall, our results suggest that strong hybrid incompatibilities between Arctic lineages may develop at very low levels of genetic divergence and thus most probably at astonishing speed, possibly within millennia after the lineages separated from a common ancestor.

As highlighted by Coughlan and Matute (2020), divergence is reversible at almost any point along the speciation continuum, although it becomes more difficult with increasing complexity of incompatibilities. Hybridization and introgression between diverging incipient species can reverse incompatibilities by replacing incompatible allelic combinations with compatible ones. Notably, seeds often disperse across vast areas in the Arctic, even in species lacking specialized morphological adaptations to long-distance dispersal (Abbott and Brochmann, 2003; Brochmann *et al.*, 2003; Alsos *et al*., 2007, 2015), providing ample opportunities for secondary contact among divergent lineages. However, secondary contact among predominantly selfing lineages will only rarely result in hybridization and introgression, rendering few possibilities for the collapse of incipient species detected in this study.

Our findings of little genetic divergence and thus probably recent separation among the populations of these species is in agreement with the long-held view that the Arctic flora is young in evolutionary terms, established only 2–3 million years ago (Hultén, 1937; Murray, 1995), as well as with phylogeographic studies (reviewed in Brochmann *et al.*, 2013; see also Eidesen *et al.*, 2013; Ikeda *et al.*, 2017). In line with our estimates for *C. bellidifolia*, the genetic structuring in the circumpolar species *Cassiope tetragona* (Eidesen *et al.*, 2007), *Kalmia procumbens* (Ikeda *et al.*, 2017) and *Vaccinium vitis-idaea* (H. Ikeda *et al*. unpubl. res.) appears to result from the last glacial period, probably within the last approx. 100 000 years. Many other Arctic species also show shallow phylogeographic structuring with few genetic groups (often inferred from AFLPs) and limited intraspecific DNA sequence divergence (Brochmann *et al.*, 2013; Eidesen *et al.*, 2013). Only few phylogeographic groups were identified in the three *Draba* species using both AFLPs and microsatellites (Skrede *et al.*, 2009), whereas numerous reproductively isolated populations were found within the same species (Grundt *et al.*, 2006), supporting rapid development of sterility barriers. In contrast, such rapid evolution of hybrid sterility appears to be infrequent in non-Arctic plants; potential examples are *Collinsia sparsiflora* (Moyle *et al.*, 2012) and *Mimulus* spp. (Sweigart *et al.*, 2006; Brandvain *et al.*, 2014; Sandstedt *et al.*, 2021).

Divergence time estimates for Arctic species may to some degree be underestimates because of latitudinal differences in rates of molecular evolution (e.g. Wright *et al.*, 2006; Gillman *et al.*, 2010; Dowle *et al.*, 2013). Drivers suggested to cause faster rates of molecular evolution at lower latitudes include direct effects of temperature on mutation rates, higher metabolic rates and shorter generation times (Rohde, 1992; Dowle *et al.*, 2013). However, the reported magnitude of the differences in rates of molecular evolution in plants is small [e.g. doubled rates in an internal transcribed spacer (Wright *et al.*, 2006) and a 51 % increase in 18S, (Gillman *et al.*, 2010) for low- vs. high-latitude plant species]. We are therefore confident that the reported latitudinal differences in rates of molecular evolution do not affect our main conclusion that sterility barriers evolve rapidly in Arctic plants.

Our results showing widespread and rapid incipient speciation in Arctic plants stand in strong contrast to a view of the Arctic as an ‘evolutionary freezer’ with low evolutionary rates and little species diversity due to extreme environmental constraints such as low temperature, a short growing season and drought (Brochmann and Brysting, 2008; Brochmann *et al.*, 2013). Rather, our results add to a growing body of evidence suggesting that both plants and animals at high latitudes may show higher speciation rates, as well as higher extinction rates, compared with the tropics (Botero *et al.*, 2014; Weir, 2014). A high rate of species turnover in the Arctic may be associated with higher environmental harshness and disturbance during the Pleistocene climatic oscillations, resulting in habitat loss and fragmentation.

### Why do hybrid incompatibilities accumulate rapidly in Arctic lineages?

It is possible that rapid and widespread evolution of incipient species in the Arctic flora is associated with some particular characteristics of this extreme environment, for example by frequent bottlenecks induced by the glacial cycles, and/or by highly selfing mating systems. Many Arctic species appear to have been heavily and successively bottlenecked during leading-edge colonization across oceans and vast areas of deglaciated terrain.

Such bottlenecking could facilitate the establishment of underdominant incompatibilities (e.g. chromosomal rearrangements; Rieseberg, 2001), especially in conjunction with inbreeding (see below). In a multispecies study covering the entire circumpolar area, Eidesen *et al.* (2013) found a gradual decrease in genetic diversity from the major glacial refugium of Beringia into deglaciated areas, resulting in low diversity in northern Europe and the Arctic Atlantic islands.

Another potential driver of the accumulation of hybrid incompatibilities is highly selfing mating systems, which are common in Arctic plants (Brochmann and Steen, 1999). Selfing provides reproductive assurance in an environment where pollinators are scarce and shows extremely weather-dependent activity, and is typically conferred by spontaneous bending of the stamens over the stigma within the bud or soon after anthesis (Brochmann, 1993; Brochmann and Steen, 1999; Brochmann and Håpnes, 2001). Notably, Arctic plants were not included in the global analysis of mating system variation by Moeller *et al.* (2017), who did not find support for increasing pollen limitation towards higher latitudes.

Our results are remarkable in that all of the five selfing species we successfully crossed showed widespread intraspecific hybrid sterility, whereas the single outcrossing species for which we were able to obtain crossing data, *Silene acaulis*, did not (Fig. 1). Although we found a slight effect of cross type for pollen fertility for the parents vs. the across-region crosses for this species, there were no effects of cross type between across-region crosses and within-population crosses. It is also notable that its congener, the selfing *Silene uralensis*, showed virtually complete hybrid sterility (Fig. 1; Supplementary data Tables S3 and S8). The data we were able to obtain for *S. uralensis* are admittedly limited, but nevertheless compelling: we examined 14 F_1_ hybrids which all showed close to zero pollen fertility and seed set, whereas the parents had high fertility. The role of mating systems in the evolution of RI has been discussed to some degree in the speciation literature. Some authors have suggested that predominant selfing may be an efficient mechanism promoting the evolution of RI because it limits gene flow among populations (Grant, 1971; Levin, 1971). Because the establishment of BDM incompatibilities is inhibited by gene flow, the strong reduction in gene flow brought about by selfing should facilitate their accumulation via either adaptation or genetic drift (Orr and Orr, 1996; Coyne and Orr, 2004). Selfing also increases the probability of fixation of recessive beneficial alleles (Charlesworth, 1992). If recessive beneficial mutations more commonly contribute to BDM incompatibilities than additive or dominant mutations, then this could increase the rate at which they accumulate. Lastly, selfing increases the fixation probability of underdominant mutations such as chromosomal rearrangements via genetic drift (Stebbins, 1971; Rieseberg, 2001). In *Draba nivalis*, the genetic analyses of post-zygotic RI were largely consistent with these predictions: single locus underdominance, a putative chromosomal translocation, and BDM and cytonuclear incompatibilities all contribute to RI (Skrede *et al.*, 2008; Gustafsson *et al.*, 2014).

### Conclusions

In this study, we show that intrinsic post-zygotic RI can develop rapidly in Arctic plant species, and that the evolution of hybrid incompatibilities is widespread across divergent lineages in the Arctic flora. It is possible that rapid and widespread evolution of incipient species is associated with some particular characteristics of the extreme Arctic environment, for example by frequent bottlenecks induced by the glacial cycles and/or by highly selfing mating systems. Thus, high extinction rates (rather than low speciation rates), possibly associated with the Pleistocene glacial cycles, are likely to contribute to the low taxonomic diversity of the contemporary Arctic flora.

## SUPPLEMENTARY DATA

Supplementary data are available online at https://academic.oup.com/aob and consist of the following. Table S1: list of all species collected in the field. Table S2: sampling data for populations used in crossing experiments and molecular analyses. Table S3: phenotype data for experimental crosses. Table S4: flow cytometric analysis of plants used in crossing experiments. Table S5: phenotype data for parental populations used in crossing experiments. Table S6: summary statistics of cross type effects on F_1_ pollen fertility and seed set. Table S7: summary statistics of cross type effects on seed production and germination success in *C. bellidifolia* and *C. groenlandica*. Table S8: summary statistics of the post-hoc Tukey pairwise comparison between pollen fertility and seed set in F_1_ hybrids. Table S9: summary statistics of the effects of distance between parental populations on F_1_ pollen fertility and seed set. Table S10: summary statistics of the family effects on F_1_ pollen fertility and seed set. Table S11: summary statistics of sequence data for *Cardamine bellidifolia*. Table S12: maximum likelihood estimates of demographic parameters between reproductively isolated populations of *Cardamine bellidifolia.* Figure S1: fluorescence histograms of species analysed for flow cytometry. Figure S2: family effects on F_1_ pollen fertility and seed set for *C. groenlandica* and *S. hyperborea.* Figure S3: hybrid sterility and genetic distances for *Cardamine bellidifolia*. Figure S4: posterior probability distribution of demographic parameters for each pair of geographic regions for *Cardamine bellidifolia.* Appendix S1: description of the six species successfully used in crossing experiments.

mcab128_suppl_Supplementary_Figure_S1

mcab128_suppl_Supplementary_Figure_S2

mcab128_suppl_Supplementary_Figure_S3

mcab128_suppl_Supplementary_Figure_S4

mcab128_suppl_Supplementary_Table_S1

mcab128_suppl_Supplementary_Table_S2

mcab128_suppl_Supplementary_Table_S3

mcab128_suppl_Supplementary_Table_S4

mcab128_suppl_Supplementary_Table_S5

mcab128_suppl_Supplementary_Table_S6

mcab128_suppl_Supplementary_Table_S7

mcab128_suppl_Supplementary_Table_S8

mcab128_suppl_Supplementary_Table_S9

mcab128_suppl_Supplementary_Table_S10

mcab128_suppl_Supplementary_Table_S11

mcab128_suppl_Supplementary_Table_S12

mcab128_suppl_Supplementary_Appendix
