# Supplementary figures and images for: Rapid evolution of post-zygotic reproductive isolation is widespread in Arctic plant lineages
Source: Ann Bot. 2021 Oct 13;129(2):171–84. doi: 10.1093/aob/mcab128 (PMC8796670; doi:10.1093/aob/mcab128)

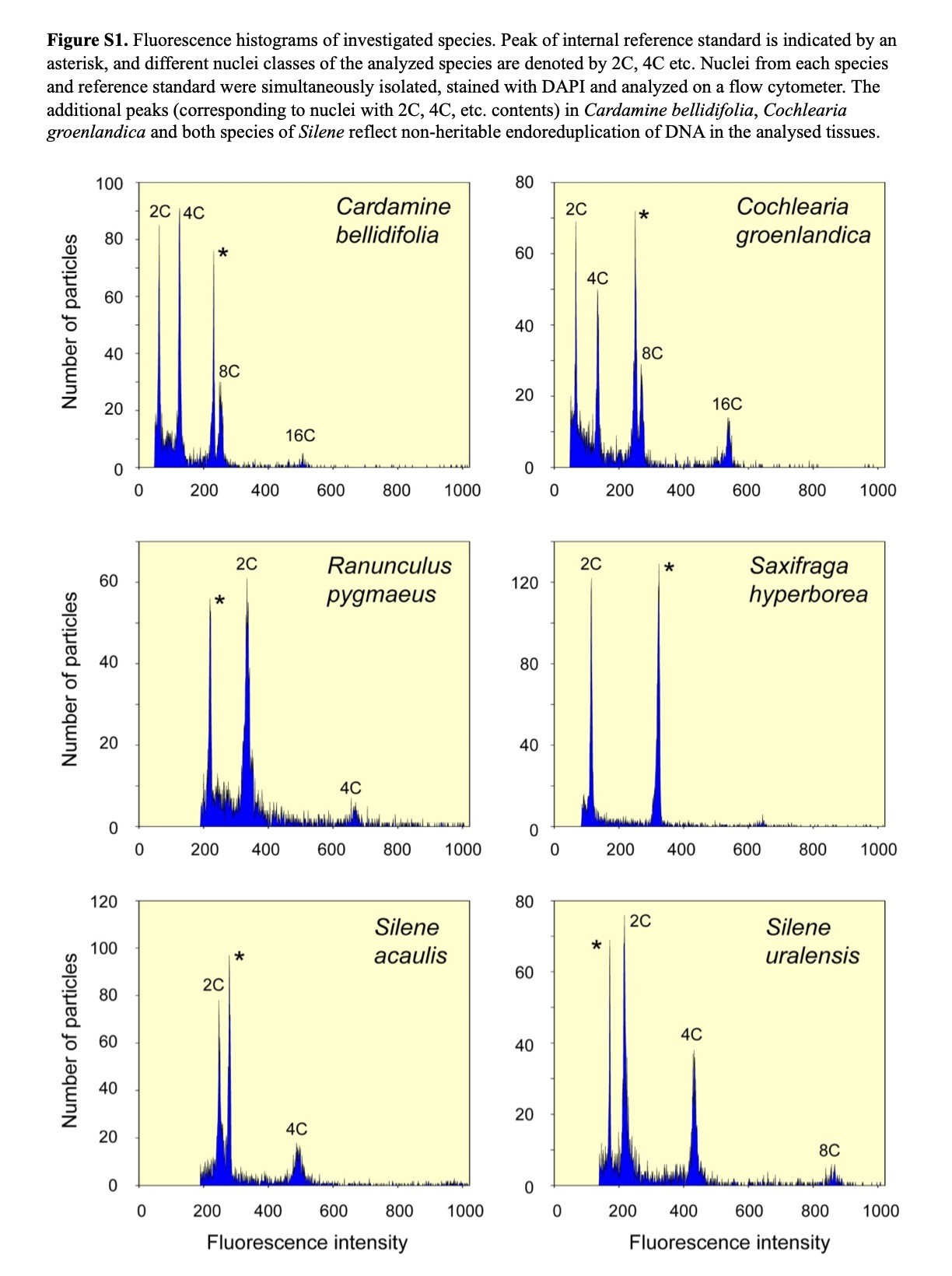

Supplement: mcab128_suppl_Supplementary_Figure_S1 [file mcab128_suppl_supplementary_figure_s1.jpeg]

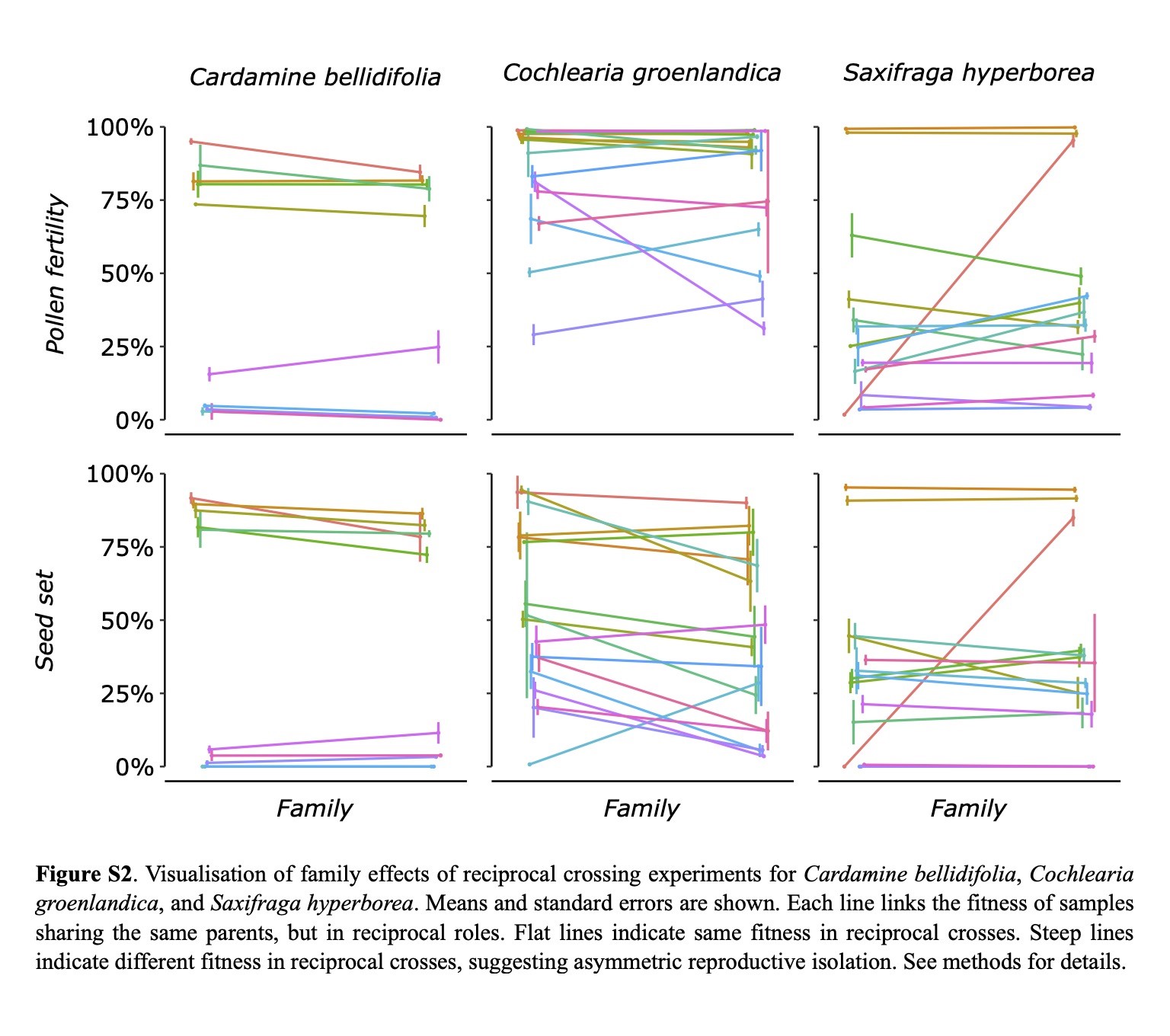

Supplement: mcab128_suppl_Supplementary_Figure_S2 [file mcab128_suppl_supplementary_figure_s2.jpeg]

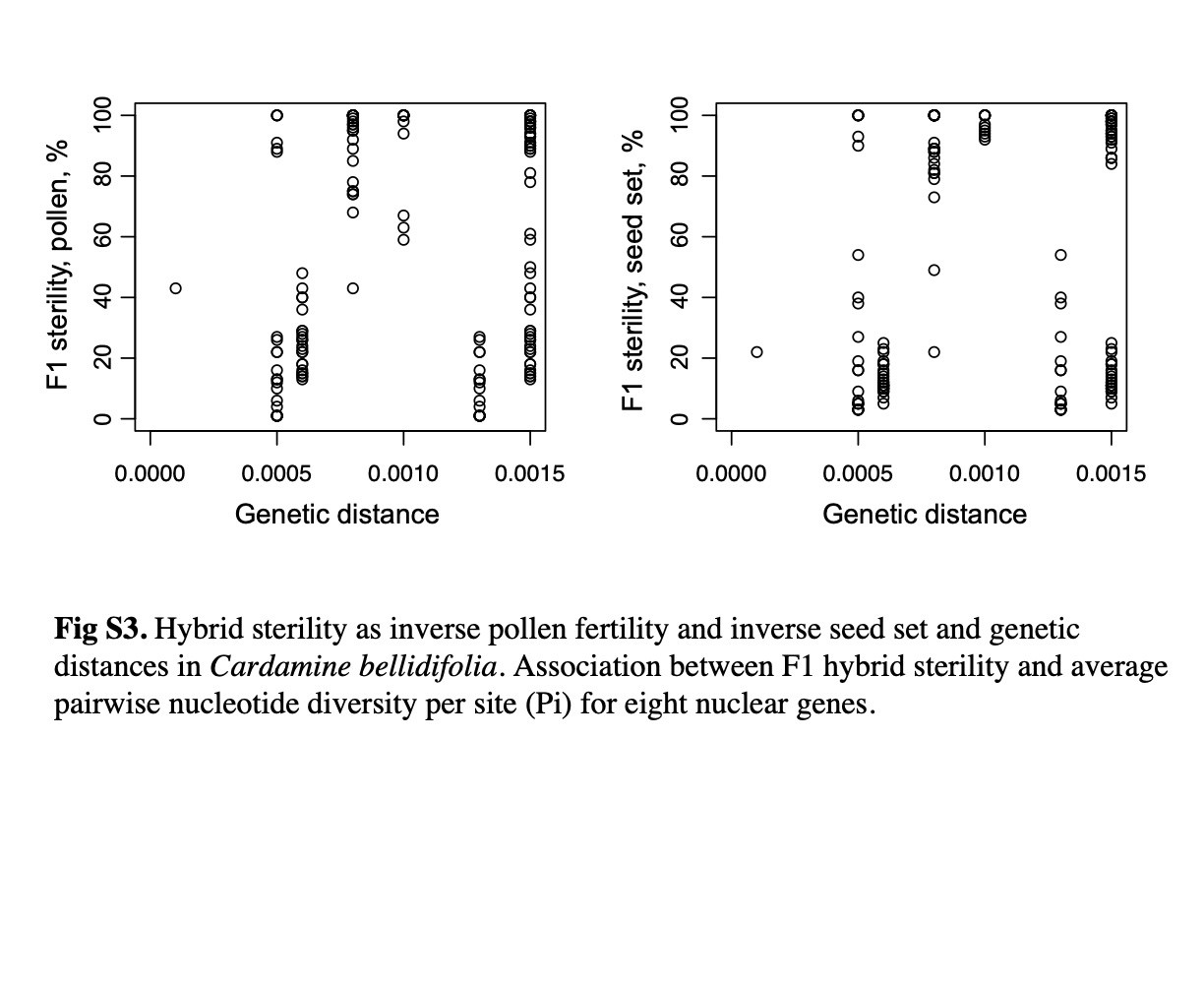

Supplement: mcab128_suppl_Supplementary_Figure_S3 [file mcab128_suppl_supplementary_figure_s3.jpeg]

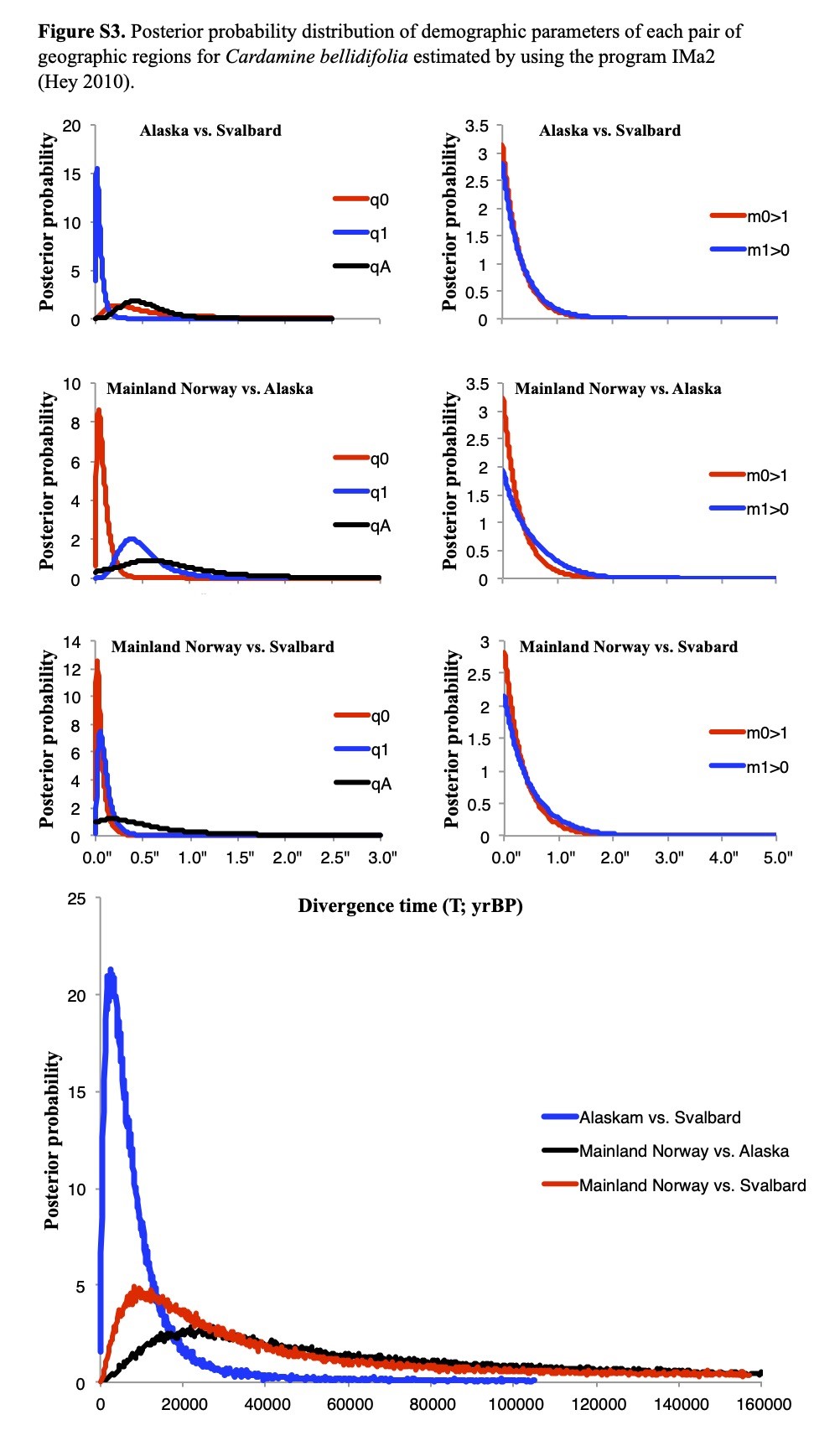

Supplement: mcab128_suppl_Supplementary_Figure_S4 [file mcab128_suppl_supplementary_figure_s4.jpeg]

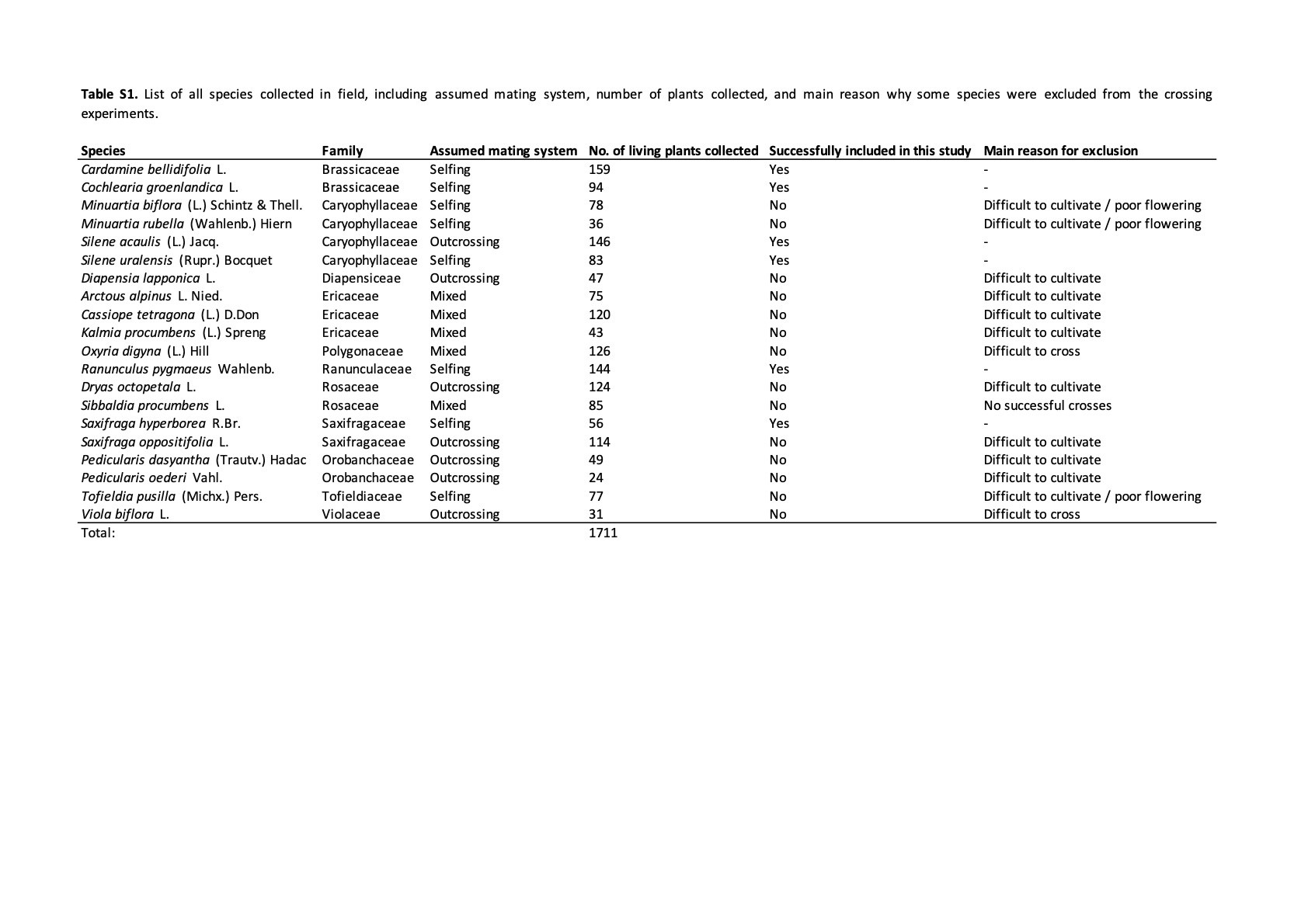

Supplement: mcab128_suppl_Supplementary_Table_S1 [file mcab128_suppl_supplementary_table_s1.jpeg]

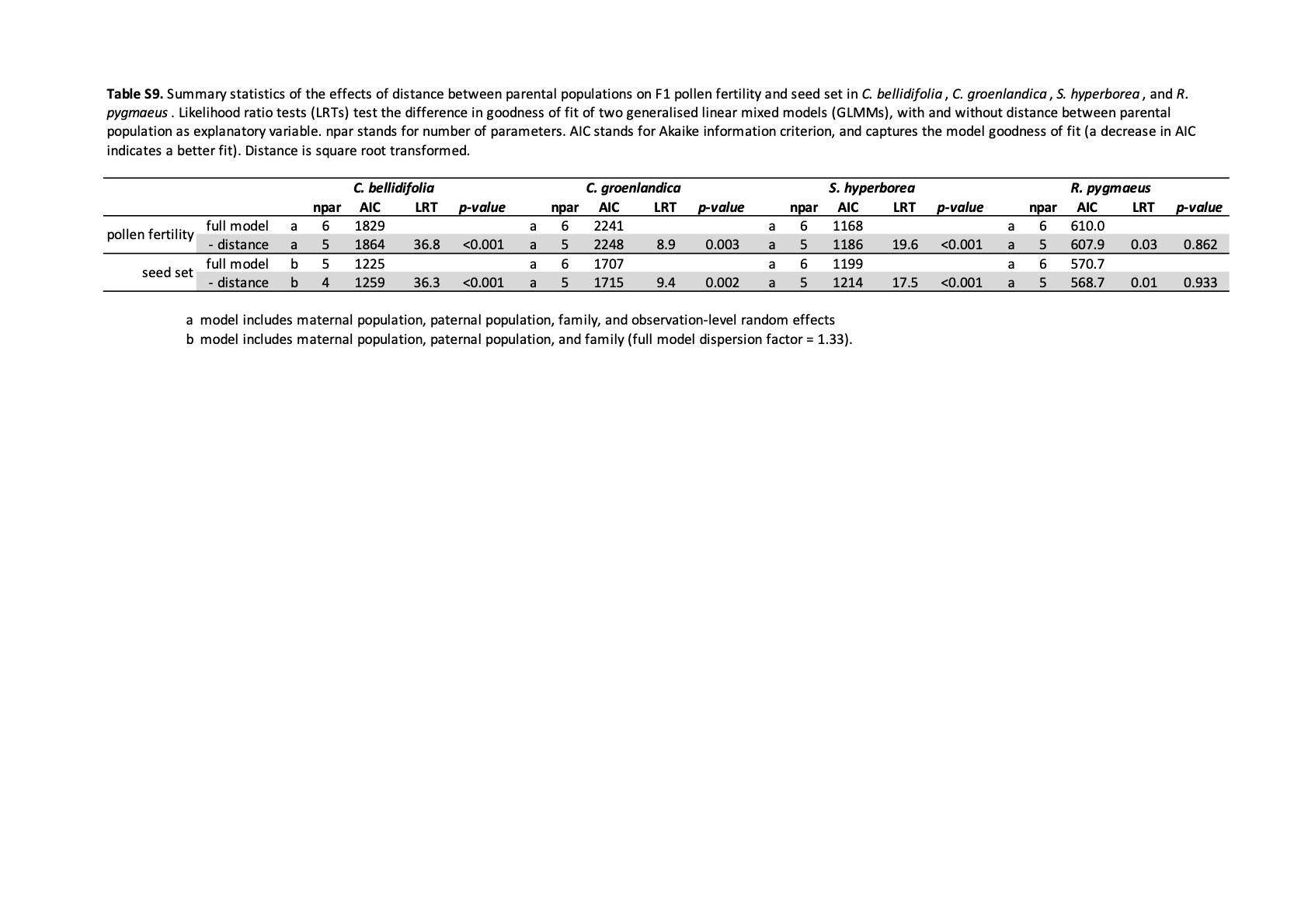

Supplement: mcab128_suppl_Supplementary_Table_S9 [file mcab128_suppl_supplementary_table_s9.jpeg]

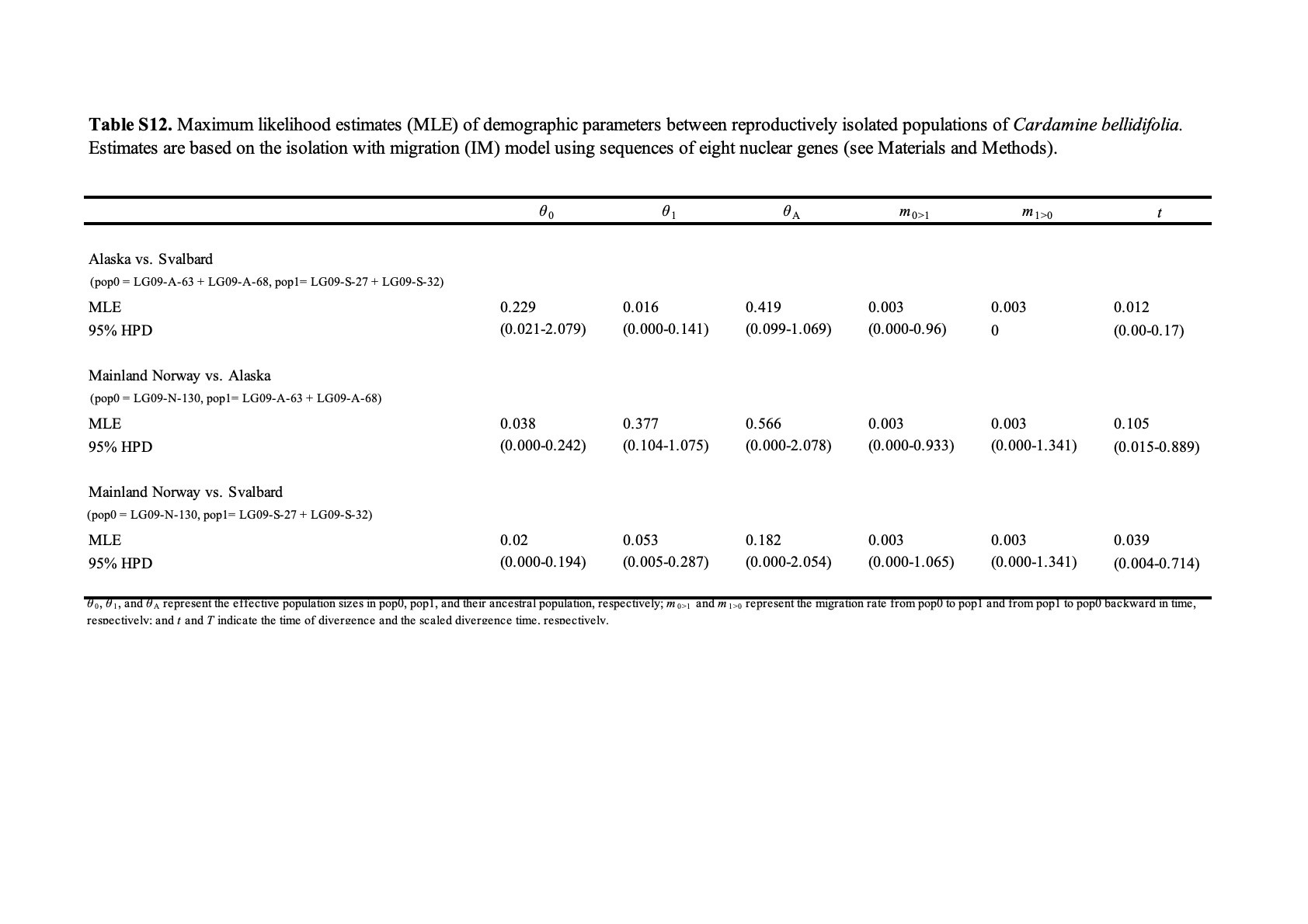

Supplement: mcab128_suppl_Supplementary_Table_S12 [file mcab128_suppl_supplementary_table_s12.jpeg]
