# Supplementary material for: Rapid evolution of post-zygotic reproductive isolation is widespread in Arctic plant lineages
Source: Ann Bot. 2021 Oct 13;129(2):171–84. doi: 10.1093/aob/mcab128 (PMC8796670; doi:10.1093/aob/mcab128)
Supplement: mcab128_suppl_Supplementary_Appendix [file mcab128_suppl_supplementary_appendix.doc]

**Appendix S1.**

**Species descriptions.** Detailed information about the six species that were successfully used in our crossing experiments.

*Cardamine bellidifolia* L. (Brassicaceae) is a small (1–10 cm) perennial herb with entire leaves in a basal rosette and a usually leafless inflorescence with small, white flowers (petals 3–4.5 mm long). It typically grows in moist, rocky areas as well as in moist tundra . Numerous reports indicate that *C. bellidifolia* is invariably diploid . It appears to be predominantly selfing; spontaneous selfing involving bending of the stamens towards its own stigma has been reported . Both fossil pollen and molecular data suggest that *Cardamine* is a fairly young genus . Although *Cardamine* has a typical short-distance mode of dispersal , *C. bellidifolia* has a complete circumpolar distribution. Its sister species is the Japanese mountain endemic *C. nipponica* ; cf. also . No infraspecific morphological variation is reported in *C. bellidifolia* in the PanArctic Flora Checklist .

*Cochlearia groenlandica* L. (Brassicaceae) is a small (1–10 cm), biennual to short-lived perennial herb with entire leaves in a basal rosette and a leafy inflorescence with small, white flowers (petals 2–4 mm long). It typically grows on tidal flats, beaches, dunes, lagoons, tundra, and bird nesting sites . *Cochlearia groenlandica* is one of the few diploid species (2*n* = 14) in an otherwise polyploid-rich genus . It has a circumpolar range and is predominantly selfing . Molecular data suggest a recent and rapid radiation of the northern European/Arctic clade of *Cochlearia*, with all current species originating in the Middle to Late Pleistocene . *Cochlearia groenlandica* is reported as polymorphic in Beringia, where a distinct species, *C. sessilifolia* Rollins, was described , but this was based on a few collections only and the reported characters were found to be within the range of *C. groenlandica* .

*Saxifraga hyperborea* R.Br. (Saxifragaceae) is a small (3–10 cm) perennial herb with palmate, 2–5-lobed leaves and often purple pigmentation, and an inflorescence sparsely to densely covered with glandular hairs and with small (petals 4–6 mm long), white to purple flowers . It lacks runners and grows in small, compact to loose tufts on damp tundra, open gravel and silt, brook and lake margins, snowbeds, shady ravines, shady cliffs, and silty and gravelly seashores . Numerous reports indicate that this circumpolar, mainly Arctic plant is invariably diploid . It is predominantly selfing, setting abundant seed (up to 100%) after spontaneous self-pollination . *Saxifraga hyperborea* belongs to the *S. rivularis* complex that has its highest taxonomic and molecular diversity in Beringia, suggesting this area as a primary Pleistocene refugium and area of diversification of the complex. Populations of *S. hyperborea* probably became separated into Pacific and Atlantic refugia during the last glaciation, with a third refugium south of the Cordilleran ice sheet in western North America . *Saxifraga hyperborea* shows some intraspecific morphological variation, and *S. flexuosa* Sternb. has been described as a distinct species growing in alpine parts of western North America north to the Brooks Range and the Richardson Mountains, and from the Russian Far East north to East Chukotka . The two species were supposed to be distinguished by the length of the flowering stem and by difference in pigmentation, but no distinction was found between them in AFLP markers . It has been suggested that the plants referred to as *S. flexuosa* are shade-growing plants belonging to *S. hyperborea*, and that the reported morphological differences are caused by phenotypic plasticity . One subspecies, *S. hyperborea* subsp. *debilis* (Engelm.) Á. Löve, D. Löve & B. M. Kapoor, has been described but from areas far south of the Arctic (Rocky Mts.); this subspecies differs in several characters and may rather represent a separate species .

*Ranunculus pygmaeus* Wahlenb. (Ranunculaceae) is a small (2–10 cm) perennial herb with deeply palmate leaves and small yellow flowers (6–7 mm broad) on peduncles that strongly elongate after flowering . It is diploid (2*n* = 16) and predominantly selfing , and usually grows in Arctic and alpine meadows and slopes, typically around persistent snow patches . The species has an Arctic-alpine distribution and is very common in the Arctic, in contrast to the Alps where it has a scattered distribution . *Ranunculus pygmaeus* shows little genetic (AFLPs and cpDNA sequences) diversity over large areas, with much lower genetic diversity in previously glaciated areas (such as the Alps and the North Atlantic region) than in areas that remained unglaciated . The lack of genetic diversity in the Alps and in the North Atlantic region apparently results from extreme bottlenecks in early phases of colonization after the last glaciation . No intraspecific morphological variation is reported in *R. pygmaeus* in the PanArctic Flora Checklist .

*Silene uralensis* (Rupr.) Bocquet (Caryophyllaceae) is a mainly diploid (2*n* = 24), perennial herb with entire leaves and a pubescent, erect peduncle (5–40 cm) bearing an erect or nodding pink to purple, sympetalous flower . It is predominantly selfing , has a circumpolar distribution, and grows primarily in dry tundra . *Silene uralensis* may have obtained its present-day distribution from unglaciated regions in Beringia . Three subspecies are reported in the PanArctic Flora Checklist . Subspecies *arctica* (Th. Fr.) Bocquet is high-Arctic and circumpolar (northernmost Alaska and Canada, N Greenland, Svalbard, Novaya Zemlya, Vaigach, N Chukotka) and distinguished from the more southern circumpolar subspecies *uralensis* by its strongly inflated calyx and emerging petals. Subspecies *ogilviensis* (A. E. Porsild) D. F. Brunt. is tetraploid and occurs in Yukon, Canada . The material used in this study belongs to ssp. *arctica* (the Svalbard material) and ssp. *uralensis* (the Alaska material).

*Silene acaulis* (Caryophyllaceae) is a small (2–5 cm), diploid (2*n* = 24), mat- or cushion- forming, long-lived herb with thin, densely crowded and imbricate leaves, and pink to purple small flowers (5–10 mm in diameter). It grows in Arctic and alpine tundra, in gravelly, often wet places and on rocky ledges . This species is gynodioecious and predominantly outcrossing . *Silene acaulis* is not fully circumpolar; there is a large gap in Siberia separating its broadly amphi-Atlantic and broadly amphi-Beringian range. The Beringian-Cordilleran plants have been reported to differ from the amphi-Atlantic ones in leaf and flower characters and proposed as a separate subspecies (ssp. *subacaulescens* (F. N. Williams Hultén), but its distinction in North America is not clear-cut , and molecular (AFLPs, cpDNA sequences) variation in the species is not concordant with the variation in morphology (Gussarova *et al*., 2015). *Silene acaulis* is genetically diverse with distinct geographical structuring, suggesting persistence in multiple glacial refugia. The main split in the AFLP variation was observed between the amphi-Atlantic and amphi-Beringian ranges .

**References:**
